# Mental model-based repeated multifaceted (MRM) intervention design: a conceptual framework for improving preventive health behaviors and outcomes

**DOI:** 10.1186/s13104-021-05516-9

**Published:** 2021-03-19

**Authors:** Mazbahul G. Ahamad, Fahian Tanin

**Affiliations:** 1 University of Nebraska-Lincoln, 140 Keim Hall, 1825 N 38th St, Lincoln, NE 68583 USA; 2 Sylhet, Bangladesh

**Keywords:** Balance test, Experimental design, Health education and promotion, Impact evaluation of health policy and social program, Intervention design, Mental model mapping, Monetary and behavioral interventions, Multifaceted intervention, Preventive health behavior and outcome, Water, Sanitation, Hygiene

## Abstract

**Objective:**

Field interventions employed to improve preventive health behaviors and outcomes generally use well-established approaches; however, recent studies have reported that health education and promotional interventions have little to no impact on health behaviors, especially in low- and middle-income countries. We aimed to develop a conceptual framework to improve intervention designs that would internalize these concerns and limitations.

**Results:**

We identified three major experimental design- and implementation-related concerns associated with mental models, including the balance between the treatment and control groups, the treatment group’s willingness to adopt suggested behaviors, and the type, length, frequency, intensity, and sequence of treatments. To minimize the influence of these aspects of an experimental design, we proposed a mental model-based repeated multifaceted (MRM) intervention design framework, which represents a supportive intervention design for the improvement of health education and promotional programs. The framework offers a step-by-step method that can be used for experimental and treatment design and outcome analysis, and that addresses potential implementation challenges.

**Supplementary Information:**

The online version contains supplementary material available at 10.1186/s13104-021-05516-9.

## Introduction

Public health intervention research generally applies existing, well-known experimental designs to improve preventive health behaviors and outcomes; however, recent literature has reported that water, sanitation, and hygiene (WASH)-related health education and promotional interventions have had little to no effect on these behaviors and outcomes [1–4]. These types of interventions may be minimally effective or ineffective due to inappropriate (or appropriate but ineffective) experimental and treatment designs, the influence of contextual factors [5], or unobserved events, especially in low- and middle-income countries [1, 2, 4, 6]. Intervention programs typically require a change in behavior among the members of a treatment group (e.g., individuals or households); this change is intended to improve preventive health behaviors and outcomes [7]. However, behavioral changes are directly associated with various factors, for example, the experimental group’s willingness to accept the suggested behaviors, their cognitive ability to adapt to new behaviors, and their mentality (or mental model) [8–11]. Mentality is particularly important because “mental models are how we understand the world. Not only do they shape what we think and how we understand but they shape the connections and opportunities that we see.” [12]. Similarly, “mental models are how we simplify complexity, why we consider some things more relevant than others, and how we reason” [12]. These factors can create major challenges to intervention design and during the implementation phases of public health research and program development. Increasing the effectiveness of interventions that focus on improving preventive health behaviors may be difficult [13] due to the complex nature and context-dependency of these behaviors [5, 14, 15].

When we reviewed interventions that focus on preventive health behaviors, we observed three common concerns among interventions that reported little to no effect on behavioral outcomes. First, these intervention approaches implicitly assumed that all treated individuals or households were identical, with similar cognitive capacities or mental models; such an assumption increases the likelihood of sample imbalance and bias in final outcome estimates [16]. The standard sample balance tests disregard behavioral or cognitive factors [17], despite frequently taking into consideration more easily observable socioeconomic and demographic characteristics (e.g., income, wealth, age) and, occasionally, knowledge level of health behaviors. Second, behavioral change programs require long-term interventions and frequent follow-ups [18], and treatment effects often wane over time, particularly after single-treatment-based interventions [19]. Third, the treatment groups’ exposure to the intervention does not vary or repeat throughout the study period, which is not comparable to the changing conditions in the real world [20].

Although some corrective measures can be applied to address the treatment-waning effect (e.g., as occurs in repeated or multifaceted interventions), no assessments that could be used to understand a treatment group’s mental model as associated with a public health intervention design are currently available. From a public health perspective, an individual’s mental model explains the experimental subject’s cognitive ability to perceive potential health risks and perform the necessary decision-making that influences health outcomes. Therefore, identifying and understanding the mental models of both the treatment and control groups is important to the design of cognitive ability- or mental model-based treatments that can be used to generate the expected intervention effects in field experiments.

The three confounding concerns that we identified can, in combination, represent significant intervention-related sources of poor health behaviors and outcomes. A potential experimental design framework aimed to improve preventive health behavior intervention designs in response to low or no intervention effects could consider the concerns associated with mental model-based balance tests, the waning of the treatment effect, and the persistence of intervention impacts. In the study, we described the development of a conceptual framework for a mental model-based repeated multifaceted (MRM) intervention design intended to improve intervention impacts by internalizing these concerns and limitations when the likely outcomes of preventive health behaviors improvement programs are assessed.

## Main text

### Materials and methods

#### Identification of key limitations and concerns

In stage one, we identified limitations and concerns regarding intervention designs and effects. First, we conducted a narrative review of published systematic reviews to identify the structure and common components of WASH-related preventive health education and promotion interventions and outcomes. Second, we reviewed field experiment-based empirical literature regarding the limitations and concerns indicated for both statistically significant and insignificant low or no intervention effects. We only considered peer reviewed systematic reviews and articles based on field experiments in resource-poor low- and middle-income countries. We used our search strategy with PubMed and Cochrane Library to source peer-reviewed articles published from January 2010 to June 2020 that used experimental field data (Additional file 1: Table S1). Finally, 86 systematic reviews and 49 empirical articles from PubMed and 129 trials from Cochrane Library were identified as useful in exploring the “common limitations” of existing experimental design approaches and intervention outcomes that were specifically mentioned in either the results and discussion or the study limitation sections, or both.

#### Framework development

**Fig. 1:**
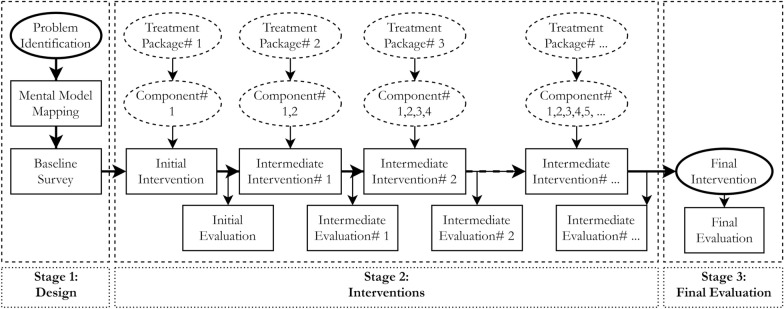
Conceptual framework for mental model-based repeated multifaceted (MRM) intervention design. The MRM framework is based on major concerns cited in existing systematic and scoping reviews, trials, and recent empirical studies regarding the approaches, frequency, treatment components, sample balance variables, and time dimensions of treatment interventions, especially WASH-related field experiments (Table 2). It considers mental model mapping to be essential and further includes two core ideas, namely, multifaceted intervention and repeated interventions, to develop this modified intervention design

Our proposed MRM intervention design framework is based on the major concerns cited in existing systematic and scoping reviews, trials, and recent empirical studies regarding design approaches, treatment frequency and components, sample balance variables, and the time dimensions of treatment interventions in published WASH-related field experiments (Table 1). At this stage, we searched the interdisciplinary literature (e.g., development economics, natural resources, behavioral economics) regarding the same concerns and limitations for interventions in public health. We then considered the different experimental approaches used in interdisciplinary fields to develop a modified intervention design that could internalize the common limitations (Fig. 1 following Additional file 2: Figure S1).

**Table 1:** Major features and concerns of preventive health behavior-related interventions

Issues	Category	Key feature	Concern/advantage
Approach	Single	Only one treatment	Treatment effect wanes over time
	Multifaceted	Multiple treatments	Persistent treatment effect
Frequency	Single intervention	One round	Treatment effect wanes over time
	Multiple intervention	Several rounds	Creates more persistent effect
Balance test	Socioeconomic and demographic factors	Income, wealth, age, sex, education	Mental model or cognitive capacity-related factors are mostly absent
Treatment component	Informational	Information-based letter	Less effective
	Educational	Education	Effective but depends on the curriculum
	Training	Hands-on experience	Effective but depends on the type and length of training
	Financial	In cash only	Attractive but ineffective if stopped
	Promotional	In kind or service	Highly effective with other treatments
	Behavioral	Weak or strong norm-based nudging	Highly effective with other financial treatments
	Mixed	Both financial and behavioral	More effective than either financial or behavioral alone
Time dimension	Short-term	Less than one year	Treatment effects wanes over time
	Medium-term	One to five years long	Better than short-term intervention
	Long-term	More than five years	Creates a more persistent effect

We reviewed water, sanitation, and hygiene (WASH)-related prevention health education and promotional interventions, (e.g., systematic reviews, empirical evidence) to explore the major features and concerns

### Results

#### Concerns related to low or no intervention effects

Based on our review of the literature and synthesis of the evidence, we found three major areas of concern in intervention design and implementation; these concerns underpin the theoretical foundation of our proposed framework. First, standard intervention design assumes that treatment subjects are similarly willing to adopt suggested preventive behaviors and have similar cognitive capacities; in fact, individuals have different cognitive capacities and mental models. These differences may lead to low-level outcomes. Human behavior is influenced by human attitudes, knowledge, perceptions, social norms, and beliefs [10, 21]; these elements together help constitute individuals’ mental models. Therefore, it is crucial to identify whether one of these elements could potentially affect intervention outcomes. Second, the impacts of single and short-term interventions wane over time [7, 19] because lasting behavioral change requires long-term intervention. Third, the responses of treatment groups to a specific intervention may vary under different or changing circumstances; most of the time, this concern has not been fully recognized. For example, a few individuals may prefer monetary incentives to behavioral nudging while unemployed. In addition, some treatment groups may respond better to behavioral nudging and hands-on experience than other groups [19, 22].

#### Conceptual framework

The MRM intervention design framework assumes mental model mapping to be essential, and it further includes two core ideas: multifaceted intervention and repeated interventions (Fig. 1). First, mapping mental models in the design stage allows the researcher or program analyst to understand a treatment group’s mental or cognitive abilities with regards to adopting suggested preventive behaviors, as well as their willingness to do so (Stage 1 of Fig. 1). Sample balance tests need to consider mental model-related variables (e.g., flexibility), along with socioeconomic and demographic variables such as age, sex, education, income, and wealth. Mapping mental models before and after each intervention is particularly important, as changes can then be identified across time and treatments.

Second, multifaceted interventions allow multiple-component treatments and can generate longer-lasting effects than single-component interventions [23]. For example, different types of educational, financial, and behavioral interventions at different intensity levels (e.g., low, standard, high) can be combined to design a treatment package (Stage 2 of Fig. 1). However, the lengths and intensities of the treatments may differ, and they should be tailored to the relevant behavior-related outcomes. In some cases, both monetary and behavioral interventions are essential in encouraging the treatment groups to adopt a behavior. Third, repeated interventions may produce more pronounced intervention outcomes (Stage 2 of Fig. 1) and are more effective than single interventions. While the impact of a single intervention wanes over time, mixed interventions with multiple rounds are more likely to produce the anticipated outcomes.

### Discussion

#### Intervention design and implementation

The MRM framework proposes a basic intervention design with three main features: mental model mapping, repeated interventions, and multifaceted interventions. In Stage 1, mapping the mental models of a treatment group before or during the baseline survey is essential to designing the initial intervention (Fig. 1). A mental model is an overall representation of an individual’s characteristics (e.g., attitudes, values, beliefs, social and cultural norms) that explains the individual’s reasoning, inferencing, and decision-making processes. These processes influence the individual’s ability to grasp, and willingness to accept suggested health behaviors [8, 9, 24–26]. Mapping mental models using modified versions of available methods [8, 26, 27] would provide insights into an individual’s or household responder’s behavioral and cognitive capacity as they relate to the adoption of the suggested health behaviors.

Stage 2 includes two different types of interventions: initial and intermediate (Fig. 1). The number of intermediate interventions, as well as their type (e.g., informational, educational, financial, behavioral) and sequence (e.g., informational-practical-behavioral, informational-behavioral-practical), should be adjusted in line with a program’s short- and long-term goals. Researchers will need to identify the appropriate length (e.g., short-, medium-, long-term) and intensity (e.g., low, standard, high) of each treatment, depending on their research goals.

In the final evaluation (Stage 3), researchers will compare the final outcomes with the baseline and intermediate outcomes to arrive at conclusions regarding specific stage-level outcomes. Redesign will be necessary if the initial treatment produces lower-than-expected outcomes.

#### Hypothetical intervention design

A hypothetical repeated multifaceted intervention design is presented in Table 2. Each component has three distinct features, which are the intervention type, length, and intensity. A standard information component can be employed in the short-term in the initial stage. In the final stage, five different components can be employed sequentially as a treatment package. This sequence could be a cluster of mixed interventions in which the order of interventions (and their close variants) is based on the mental models of the treatment group members and expected outcomes from the programs. Thus, individuals with limited learning or adoption capacity, for instance, could be treated with higher intensity.

**Table 2:** An example of a repeated multifaceted intervention design

Round	Intervention Component					
	Feature	Comp. #1	Comp. #2	Comp. #3	Comp. #4	Comp. #5
Initial	Type	Informational				
	Length	Short-term				
	Intensity	Standard				
	Evaluate	With baseline				
Int. #1	Type	Practical	Behavioral			
	Length	Short-term	Medium-term			
	Intensity	Standard	Low-level			
	Evaluate	With baseline and initial interventions				
Int. #2	Type	Informational	Practical	Financial	Promotional	
	Length	Short-term	Medium-term	Short-term	Short-term	
	Intensity	Minimum	Low-level	Standard	High-level	
	Evaluate	With baseline, initial, and intermediate #1 interventions				
Final	Type	Informational	Practical	Behavioral	Financial	Promotional
	Length	Short-term	Medium-term	Short-term	Short-term	Short-term
	Intensity	Low-level	High-level	Low-level	Standard	Low-level
	Evaluate	With baseline, initial, intermediate #1, and intermediate #2 interventions				

*Int.* Intermediate, *Comp*. Component

#### Outcome analysis

As each intervention combines multiple treatments, researchers should consider all the treatments at a given stage as a treatment package (e.g., treatments 1, 2, and 3 in combination are a treatment package for intermediate intervention #2). Comparing the outcomes of each intervention with previous interventions (e.g., comparing intermediate intervention #2 with the baseline and with intermediate intervention #1) will be necessary to reveal whether the effects of a treatment package have persisted. If a promotional component is included as a treatment, the possibility of courtesy bias [1] on the part of the responders during after-intervention data collection should be accounted for to minimize the bias in outcome estimates.

Most importantly, researchers will need to check the mental model after each intervention to compare it with the initial mental model, the subject’s willingness to accept the behavioral change (as stated in a baseline survey), and the subject’s actual or demonstrated willingness to accept the suggested behaviors. Sub-group analysis is essential to assess the adherence to suggested behaviors by different groups within or between treatment groups. A crossover design would allow for various evaluation techniques, such as a quasi-experimental design (e.g., pre-post) for the initial intervention and an experimental design (e.g., difference-in-difference) for the intermediate and final interventions.

#### Design, implementation, and analysis challenges

First, individual or household responder-level mental models vary contextually; therefore, a suitable mental model mapping and classification procedure needs to be adopted that takes the prevailing experimental contexts into consideration. A professional behavioral profiler is needed to ensure accuracy, as typical enumerators are not trained to perform mental model mapping. Second, identification of the appropriate lengths and intensities of different treatments will be challenging during the initial and first intermediate stages due to various contextual factors. Researchers could use these two stages to test the initial treatments and identify appropriate treatment conditions to employ in the later stages. Third, the effect size of intermediate interventions may be misleading due to a variety of outside factors (e.g., unexpected bad or good weather).

### Conclusion

Mental model mapping can reveal a treatment group’s mental or cognitive abilities to adopt the suggested preventive behaviors during a multifaceted program intervention. Therefore, the inclusion of a mental model-related variable as a part of the balance test is critical to understanding the cognitive ability and willingness-related balances between different treatment and control groups. It is also important to consider the various types, lengths, frequencies, intensities, and sequences of treatments to design repeated multifaceted interventions and to consider mental model mapping with the intention of improving the program effectiveness of preventive health behaviors and outcome-related interventions.

## Limitations

Our proposed framework is designed to provide an intervention model to improve health education and promotional intervention programs. An evaluation of actual effectiveness of field experiments using this framework is essential to examining our hypotheses and advancing our understanding of how MRM design can be applied to improve preventive health behaviors and outcomes.

## Supplementary Information

**Additional file 1: Table S1.** Literature search strategy for MRM intervention design, 2010–2020.

**Additional file 2: Figure S1.** Steps in identification of key concerns and framework development.

## Data Availability

Not applicable.

## Abbreviations

MRM: Mental model-based repeated multifaceted
WASH: Water, sanitation, and hygiene
