# Supplementary material for: Mental model-based repeated multifaceted (MRM) intervention design: a conceptual framework for improving preventive health behaviors and outcomes
Source: BMC Res Notes. 2021 Mar 19;14:103. doi: 10.1186/s13104-021-05516-9 (PMC7977269; doi:10.1186/s13104-021-05516-9)
Supplement: Supplementary file 1 — Additional file 1: Table S1. Literature search strategy for MRM intervention design, 2010–2020. [file 13104_2021_5516_MOESM1_ESM.docx]

**Table S1. Literature search strategy for MRM intervention design, 2010—2020.**

| **Database** | **Query** | **Results** |
| --- | --- | --- |
| PubMed | Search: (((water) AND (sanitation)) AND (hygiene)) AND (WASH)  Filters: Meta-Analysis, Review, Systematic Review, in the last 10 years | 80 |
|  | Search: ((((water) AND (sanitation)) AND (hygiene)) AND (WASH)) AND (behavioral change)  Filters: Systematic Review, in the last 10 years | 6 |
|  | Search: ((((water) AND (sanitation)) AND (hygiene)) AND (WASH)) Filters: Randomized Controlled Trial, in the last 10 years | 49 |
|  | Search: ((((water) AND (sanitation)) AND (hygiene)) AND (WASH)) AND (mental)) Filters: Randomized Controlled Trial, in the last 10 years | 0 |
|  | Search: ((((water) AND (sanitation)) AND (hygiene)) AND (WASH)) AND (mental mode)) Filters: Randomized Controlled Trial, in the last 10 years | 0 |
| Cochrane Library | (water):ti,ab,kw AND (sanitation):ti,ab,kw AND (hygiene):ti,ab,kw AND (WASH):ti,ab,kw (Word variations have been searched)" with Publication Year from 2010 to 2020, in Trials (Word variations have been searched) | 129 |
|  | (water):ti,ab,kw AND (sanitation):ti,ab,kw AND (hygiene):ti,ab,kw AND (WASH):ti,ab,kw AND (mentality):ti,ab,kw (Word variations have been searched)" with Publication Year from 2010 to 2020, in Trials (Word variations have been searched) | 0 |
|  | (water):ti,ab,kw AND (sanitation):ti,ab,kw AND (hygiene):ti,ab,kw AND (WASH):ti,ab,kw AND (mental model):ti,ab,kw (Word variations have been searched)" with Publication Year from 2010 to 2020, in Trials (Word variations have been searched) | 0 |
