# Supplementary figures and images for: Mental model-based repeated multifaceted (MRM) intervention design: a conceptual framework for improving preventive health behaviors and outcomes
Source: BMC Res Notes. 2021 Mar 19;14:103. doi: 10.1186/s13104-021-05516-9 (PMC7977269; doi:10.1186/s13104-021-05516-9)

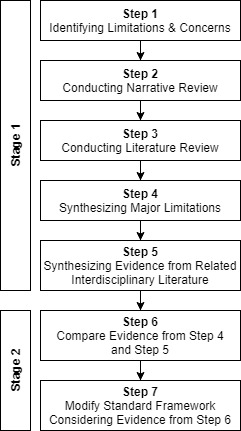


**Figure S1.** Steps in identification of key concerns and framework development.

Supplement: Supplementary file 2 — Additional file 2: Figure S1. Steps in identification of key concerns and framework development. [file 13104_2021_5516_MOESM2_ESM.docx]
